# The advances and new technologies for the study of mitochondrial diseases

**DOI:** 10.1590/S1679-45082016MD3561

**Published:** 2016

**Authors:** Bianca Bianco, Erik Montagna

**Affiliations:** 1 Faculdade de Medicina do ABC, Santo André, SP, Brazil.

**Keywords:** Mitochondria, DNA, mitochondrial, Mitochondrial diseases, Preimplantation diagnosis

## Abstract

Genetic mitochondrial disorders are responsible for the most common inborn errors of metabolism, caused by mutations in either nuclear genes or in mitochondrial DNA. This article presents the prokaryotic origin of the organelle and the relation between nuclear and mitochondrial genomes, as well as current evolutionary models for such mechanisms. It also addresses the structure of mitochondrial genes, their expression pattern, clinical features of gene defects, risk of transmission and current techniques to avoid these events in assisted human reproduction. Finally, it discusses the ethical implications of these possibilities.

## RESUMO

As doenças genéticas mitocondriais são responsáveis pelos erros inatos do metabolismo mais comuns, causados por mutações tanto em genes nucleares como no DNA mitocondrial. Este artigo apresenta a origem procariótica dessa organela, e a relação entre os genomas nuclear e mitocondrial, bem como modelos evolutivos correntes para esses mecanismos. Também trata da estrutura dos genes mitocondriais, seu padrão de expressão, características clínicas de defeitos genéticos, riscos de transmissão e técnicas atualmente utilizadas para evitar esses eventos em reprodução humana assistida. Finalmente, discute as implicações éticas dessas possibilidades.

## INTRODUCTION

Mitochondria are organelles responsible for oxidative phosphorylation by channeling electrons through the respiratory chain complexes that generate adenosine triphosphate (ATP), a nucleotide responsible for the storage of energy from cell respiration in its chemical bonds. A remarkable feature is that they possess a distinct genome of their own. Mitochondrial DNA (mtDNA) has several unique characteristics that are different from those of the nuclear genome, such as a compact, double-stranded circle (16,569bp) with its own genetic code. It contains 37 genes, 13 of which encode OXPHOS subunits, 22 tRNA genes, and 2 rRNA genes. Mitochondrial DNA contains no introns, several overlapping genes and incomplete termination codons. Maintenance of two separate genomes is a costly situation for the cell. Maintenance and expression of the few remaining mitochondrial genes require about 250 proteins encoded by the nuclear genome. There is evidence that mitochondrial sequences are copied to the nuclear genome. Even though few proteins are encoded by the mitochondrial genome, more than one thousand mitochondrial proteins are encoded by the nuclear genome.^(1)^

There is no doubt that the mitochondrial genome is of bacterial origin, specifically alpha-proteobacterial, and there is wide acceptance of a xenogenous/exogenous (endosymbiotic) origin of the mitochondrion, instead of the autogenous/endogenous (non-symbiotic) origin hypotheses. It is inferred that proteins encoded by the mitochondrial genome, as well as nucleus-encoded mitochondrial proteins, were genes transferred to the nucleus by endosymbiotic gene transfer (EGT) during mitochondrial evolution.^(2)^

Mitochondrial disorders are the most common inborn errors of metabolism, affecting >1 in 7,500 live births, and are caused by mutations in either nuclear genes or mtDNA; the latter is only maternally transmitted. Mitochondrial disorders may cause miscarriage and stillbirth; death in infants, children, and young adults; or severe symptoms with an onset in adulthood. Clinical manifestations may present in just a single affected tissue or organ, but a multisystemic or multiorgan involvement is more common and has the greatest effects on organs with a high energy demand.^(3)^ Since patients can manifest it at any age, with almost any affected body system, and symptom severity can also vary widely, recognition and diagnosis of mitochondrial diseases are difficult and clinicians are often left with more questions than answers for families. Genetic counseling is challenging considering the uncertainties about prognosis and risk of recurrence.^(4)^

All mtDNA molecules within a cell can be identical (homoplasmy), whereas two or more types of mtDNA molecules that differ in sequence can coexist in the same cell, tissue, or even in the same organelle (heteroplasmy), ranging between zero and 100%. Clinical manifestations are partly related to the mutation load and manifest when the mutant load (proportion of mutant mtDNA) exceeds a threshold of expression. Disease-causing mutations in mtDNA can be due to large rearrangements, point mutations, or a reduced copy number, in some cases leading to extensive depletion of mtDNA.^(3)^

The recurrence risk depends on the nature of the underlying primary genetic defect: large, single mtDNA deletions have a low recurrence risk of 1 in 25 in female patients and zero in males,^(5)^ but multiple mtDNA deletions due to autosomal recessive or dominant nuclear gene defects have a recurrence risk of 25% or 50%, respectively, for other siblings. Multiple mtDNA deletions, occurring as somatic mutations as part of the normal aging process, are not related to the clinical phenotype and do not recur. In addition, it should be noted that mtDNA point mutations can be very frequent,^(6)^ especially at low levels of heteroplasmy, which implies that these mutations do not necessarily cause the clinical symptoms. Homoplasmic disease-causing point mutations in mtDNA are also known, generally causing less severe disease or with reduced penetrance.^(3)^ The difficulty in accurately predicting the clinical consequences and inheritance of mtDNA abnormalities is much more complex owing to several complicating factors, including heteroplasmy and the threshold effects.^(4)^

Patients with clinically suspected mitochondrial disease would be confirmed through respiratory chain dysfunction and/or biochemical abnormalities, and these many may not have yet a known molecular etiology. Although genetic testing technology is advancing rapidly leading clinicians to offer more targeted genetic counseling to families, and genomic medicine has an increased ability to detect genetic alterations, uncertainties about pathogenicity, prognosis, cognitive limitations, and life span often remain elusive for families at risk. A genetic counselor is poised to explain the aforementioned complexities of mitochondrial disease prognosis, inheritance, and reproductive testing options in a tailored, family-centered approach.^(4,7)^

The transmission of mtDNA disorders can be prevented by several approaches, such as oocyte donation and prenatal tests. Preimplantation genetic diagnosis or nuclear genome transfer apply to different situations (*de novo* disease *versus* familial disease; deletions *versus* point mutations), each with specific advantages and disadvantages and technical and ethical constraints.^(8)^ Preimplantation genetic diagnosis can be offered to select unaffected embryos generated by *in vitro* fertilization. Preimplantation genetic diagnosis for mtDNA diseases is technically easier; however, heteroplasmic mtDNA mutations require case-by-case counseling, considering the uncertainties linked to this risk-reduction strategy.^(9)^ Nuclear genome transfer or mitochondrial donation involve the transfer of the nuclear genome from an oocyte with mutated mtDNA in the cytoplasm (donor) to an enucleated acceptor oocyte of a healthy donor (acceptor) with presumably normal, mutation-free mtDNA.^(3,10)^ Consequently, the offspring would not carry the mtDNA mutation present in the mother and would not suffer from the familial mtDNA disease. Thus, the generated offspring will present with three distinct genetic information patterns: paternal genome, maternal nuclear genome, and mitochondrial genome of a donor.

In February 2015, the United Kingdom Parliament voted in favor of clinical application of *in vitro* fertilization procedures involving mitochondrial donation and became the only region in the world to legalize germ-line technologies.^(11)^ Two important questions were raised especially about the role of mtDNA and the interactions between mtDNA and nuclear DNA. The first was whether the embryo was at risk if there was a mismatch between the mtDNA haplotype of the mitochondria donor and that of the intending mother, and the second one was whether some of the faulty mitochondria would remain attached to the nucleus during the process of transfer. However, at the request of the Department of Health, the Human Fertilisation and Embryology Authority conducted three scientific reviews, and a panel of experts reported on and assessed evidence about the safety and efficacy of mitochondrial replacement techniques. Clearly, many safety, ethical, regulatory, and legal issues that apply to nuclear genome transfer must be considered.^(8)^
